# Evaluation of the internet-based intervention *“Selfapy”* in participants with unipolar depression and the impact on quality of life: a randomized, parallel group study

**DOI:** 10.1007/s11136-024-03606-2

**Published:** 2024-02-25

**Authors:** Cora Schefft, Rico Krämer, Raoul Haaf, David Jedeck, Anna Schumacher, Stephan Köhler

**Affiliations:** 1 Department of Psychiatry and Neurosciences, Charité – Universitätsmedizin Berlin, Campus Mitte, Berlin, Germany; 2 Department of Psychology, Humboldt University of Berlin, Berlin, Germany; 3 Department of Psychology, Sigmund-Freud Privat Universität, Berlin, Germany

**Keywords:** Unipolar depression, Internet-based intervention, Blended treatment, Quality of life, Randomized controlled trial, Cognitive behavioral therapy

## Abstract

**Purpose:**

Depressive disorders cause a major burden of disease worldwide and often lead to a loss of social functioning. Patients suffering from depressive disorders report a lower quality of life (QOL) than people without a history of mental health issues. Internet-based interventions (IBIs) based on cognitive behavioral therapy (CBT) are effective in reducing symptom severity but data on their impact on quality of life in clinically depressed patients so far is scarce.

**Methods:**

*Selfapy* is a CBT-based IBI for depressive disorders. 401 participants (332 female, mean age 37 (*SD* = 11) with a diagnosis of major depressive disorder (MDD) or dysthymia were enrolled in a randomized, parallel, three-arm trial comparing a therapist-guided *Selfapy* intervention with an unguided *Selfapy* intervention and a waiting list control. QOL was measured using the WHOQOL-BREF at baseline, post-treatment (12 weeks) and at 24-week follow-up. The effects of the interventions on QOL were calculated using linear mixed effects models.

**Results:**

At post-treatment (12 weeks) the guided and unguided intervention groups reported an increase in QOL on physical and psychological health domains compared to controls (significant group*time interaction). The gain in QOL was maintained over the follow-up period only for psychological health. QOL decreased in the social relationships and environment domains over the course of treatment and during the follow-up treatment for all participants. There were no differences between the guided and the unguided intervention.

**Conclusion:**

*Selfapy* proved to positively affect psychological and physical QOL in a sample of participants suffering from depressive disorders and can therefore be considered an effective and highly scalable therapeutic tool. The pattern of results might partly be attributable to effects of the COVID-19 pandemic and public health measures that coincided with the trial.

*Trial registration*: German Clinical Trials Register (DRKS): DRKS00017191. Registered June 14th, 2019, https://www.drks.de/drks_web/navigate.do?navigationId=trial.HTML&TRIAL_ID=DRKS00017191.

**Supplementary Information:**

The online version contains supplementary material available at 10.1007/s11136-024-03606-2.

## Plain English summary

Mobile psychotherapeutic interventions are important to meet the increasing demand for help in patients with depressive disorders. In our article, we report the results of a study investigating the effect of a therapeutic app (*Selfapy*) on the quality of life of participants with depression. Quality of life is an important measure since it maps not only freedom of symptoms but also long-term recovery. Here, we compared a therapist-guided group with an unguided group and a control group that received the treatment after a waiting period of 24 weeks. We were able to show that over the duration of the *Selfapy* course both the guided and the unguided groups improved their physical and psychological quality of life compared to the control group. The gain in psychological quality of life was maintained for another 12 weeks thereafter. However, social relationships and environmental quality of life were perceived as progressively worse by all groups over the same period, which could partly be explained by the effects of the simultaneous spreading of the COVID-19 pandemic. In conclusion, the *Selfapy* app is well suited to help alleviate depressive symptoms and improve aspects of quality of life and is therefore an important addition to the treatment options for depressive disorders.

## Background

With 350 million people suffering worldwide, depression is one of the most commonly occurring disorders [1]. In addition to psychological impairments, depressive disorders can affect a person’s physical health and their degree of social and professional functioning. Moreover, depressive disorders are associated with numerous psychiatric and physical comorbidities such as anxiety and substance-related disorders as well as cardiovascular and metabolic conditions [2]. Beyond these aspects of mental and physical health, and social functioning, depression is associated with low quality of life [3, 4]. The relationship between quality of life and depressive symptomatology can be considered reciprocal, and improvement in one can ameliorate the other and vice versa [5, 6]. On the one hand, subjects suffering from mental health issues and severe mental illness report *objectively* less advantageous living conditions [7, 8, 9]. On the other hand, depressive symptomatology decreases *subjective* general and (mental) health-related quality of life ratings in usually healthy individuals, irrespective of their actual lifestyle [9]. The concept of quality of life is defined by the WHO as “the individual’s perception of their position in life in the context of the culture and value systems in which they live and in relation to their goals, expectations, standards, and concerns” [10]. Given its comprehensive scope and its emphasis on subjective evaluation, quality of life by this definition is an important outcome criterion beyond mere symptom control when it comes to assessing treatments for depression. Arguably, it is the most important one [5, 7] and its role in predicting relapse or recovery is empirically understudied [11, 12].

Both pharmacological and psychological interventions for depressive disorders have been shown to improve quality of life in the short and longer term [7, 13]. Focusing on the effects of psychotherapy of depression, a meta-analysis on 44 studies and 5264 participants found moderate effects on quality of life (*g* = 0.33, 95% CI [0.24, 0.42]), while in a subsample of participants carrying a clinical diagnosis of major depressive disorder (MDD) the effect was more pronounced than in those without diagnoses of depression (*g* = 0.49; 95% CI [0.36, 0.61] vs. *g* = 0.23; 95% CI [0.13, 0.34]) [6].

Due to their widespread use, internet-based interventions (IBI) have become a way of making mental health-related content available to a large number of users worldwide. Numerous IBIs have been launched in recent years [14]. However, only a fraction of these interventions have undergone empirical testing so far [15]. Meta-analyses have shown that IBIs with varying levels of guidance have small to moderate, sometimes large effects on depressive symptoms, depending on the control group [15, 16, 17, 18].

IBIs for depressive disorders often adopt elements of cognitive behavioral therapy (CBT) since it is not only one of the most effective antidepressant treatments but also very suitable for IBIs, given its highly structured and modular form and its focus on homework between sessions [19]. In a 2019 meta-analysis examining the effects of IBIs on depression and anxiety symptoms in mixed samples, about half of the 66 pooled trials applied some forms of cognitive and/or behavioral approaches [17]. Effect sizes on depressive symptoms in this meta-analysis were small to moderate, with therapist-guided interventions being associated with larger effect sizes than unguided interventions.

A recent narrative review focusing on CBT-based IBIs for depression largely did not show beneficial effects on quality of life or related concepts [20]. However, the majority of the reviewed studies included participants with elevated symptoms but not clinical diagnoses. To date, data on the effects of CBT-based IBIs on quality of life in clinical samples of people suffering from MDD are scarce. Quality of life has so far either not been included as an outcome measure or the included participants did not carry a diagnosis of MDD [20, 21].

Therefore, further research is necessary to examine the effects of IBIs on quality of life both in the short and long term. The aim of our study was to test the effectiveness of the internet-based self-help intervention *Selfapy* on quality of life in a randomized, controlled parallel group study comparing a guided form of the intervention to an unguided form and a control group (waitlist). Quality of life was operationalized as the short version of the World Health Organization Quality of Life scale (WHOQOL-BREF; [22]). WHOQOL-BREF scores were taken at baseline, at the end of the intervention (12 weeks), and at a follow-up date 24-week post-baseline assessment.

### Main hypotheses

1. Quality of life scores will increase over the course of the intervention and will be higher in the intervention groups than in the control group at the end of the intervention. The increase will be larger in the guided compared to the unguided intervention.

2. The increase in quality of life scores in the intervention groups will be maintained over the follow-up period.

## Methods

A detailed account on the study’s rationale, the intervention, and its methods are available in the published protocol and the published primary outcomes [23, 24]. The study was a parallel single-center, multi-arm trial with a waitlist control. It was approved by the ethics committee of the medical faculty of the Charité University Medicine Berlin (EA/047/19) and was conducted in line with the Helsinki Declaration of 1975, as revised in 2008 [25].

### Recruitment

401 participants from across Germany were recruited through the *Selfapy* website, which hosted a registration form for participation in the trial (www.selfapy.de/studie). We advertised the trial and its respective website on social media platforms and in information brochures of both health insurance companies and the Department of Psychiatry and Neurosciences of the Charité University Hospital Berlin. Recruitment took place between May of 2019 and January of 2021.

### Inclusion and exclusion criteria

Participants were included if they were fluent in German, between 18 and 65 years of age, met diagnostic criteria of a depressive episode or dysthymia as ascertained by Mini-International Neuropsychiatric Interview (MINI, [26]) (ICD-10: F32; F33; F34) and scored at least 13 points on the Beck Depression Inventory (BDI-II) [27]. Furthermore, uninterrupted internet access was required. Subjects were excluded if they had a primary diagnosis or a history of a psychotic disorder or bipolar disorder or were experiencing psychotic symptoms, were acutely at risk for suicide, or were experiencing suicidal ideation, were diagnosed with a substance use disorder or experiencing withdrawal symptoms. The inclusion and exclusion criteria were assessed in telephone interviews prior to inclusion. All MINI interviews were conducted by the same interviewers, a clinical psychologist and a doctoral student who were blind to the participants’ allocation. The interviewers underwent prior interview training supervised by a senior physician at the Charité Department of Psychiatry and Neurosciences.

Informed consent was obtained from all subjects for participation in the study as well as the use of their data in primary and secondary analyses. Subjects in all groups were free to continue or to seek psychological or medical help. All concurrently used treatments were ascertained repeatedly through self-report.

### Randomization and blinding

Participants who meet the inclusion criteria were randomly assigned to one of three groups in a 3:3:2 ratio: (a) immediate access to the guided depression course of *Selfapy*, (b) immediate access to the unguided depression course of *Selfapy*, or (c) access to the depression course *Selfapy*, guided, or unguided after 24 weeks (control group). Interviewers were blind to the allocation of subjects.

### Intervention

In brief, the 12-week online course *Selfapy* consists of six core modules and six optional in-depth modules mainly based on CBT elements. Within the three-arm trial, participants in the guided intervention group were given additional personal guidance by psychotherapists in training. The therapists had calls of 25–30 min duration with the participants on a weekly basis after getting to know them personally at the beginning of the trial. During the initial 30–50-min encounter, therapists introduced the functions and aims of the program and then identified the individual participant’s goals. Furthermore, the therapists introduced themselves and their background briefly and asked participants how they had gotten to participate in the study as a means to establish rapport. The therapists were kept consistent throughout the trial for each participant.

In the phone calls, the conversations revolved around the most recent course content and exercises. Personal resources and behavioral activation were addressed with the therapists putting individual emphasis on exercises.

In the unguided version, the participants followed the program independently with a chat function available to them in order to pose questions regarding the correct use of the course. In order to adhere to the safety concept, a psychologist also monitored the participants’ self-report measures in order to react to signs of suicidality or self-harm.

Subjects in the waiting list control group did not receive treatment or support through the trial team during the waiting period; however, they were free to seek treatment outside of the trial. During the waiting period, they received several emails containing instructions for non-specific mood-stabilizing exercises and techniques such as body scanning, guided abdominal breathing or guided imaginations in order to increase adherence. After 24 weeks of waiting, participants in this condition received access to the *Selfapy* course. They were free to choose between the guided or the unguided version.

### Survey instruments

This publication reports results of the secondary outcome measure quality of life. The WHOQOL-BREF [22] is a widely used self-report questionnaire measuring quality of life on a 1 to 5 Likert scale in four domains:

- Physical Health: Pain and discomfort, sleep and rest, energy, activities of daily living, mobility, dependence on medicinal substances and medical aids, work capacity.
- Psychological Health: Positive feelings, cognitive abilities, self-esteem, body image and appearance, negative feelings, and spirituality/religion/personal beliefs.
- Social Relationships: Personal relationships, social support, and sexuality.
- Environment: Freedom, physical safety and security, home environment, finances, health and social care, opportunities for acquiring new information and skills, participation, transport, and physical environment.

With its 26 items, its length is suitable for both clinical trials and clinical practice. Higher scores indicate a higher quality of life, scores range from 0 to 100. Its German version shows high internal consistency for the general population and clinical populations (Cronbach’s α between 0.72 and 0.83) [28].

We measured outcomes at three different timepoints: (1) at the start of the intervention, (2) 12 weeks after the start of the intervention (end of the intervention), and (3) 12 weeks after the end of the intervention (24-week naturalistic follow-up).

### Statistical analysis

All analyses were performed in R (Version 4.2.1) [29]. Means are reported with their respective 95% confidence intervals (95% CI) or standard deviations (*SD*), and medians with their respective interquartile range (IQR). Two-sided tests were used with a 0.05 significance level.

Analyses followed an intention-to-treat (ITT) approach. Missing data at 12 weeks were imputed by multiple imputations by chained equations which is a suitable method for handling missing data in randomized controlled trials [30]. We assumed data to be missing at random (MAR) and evaluated our assumption in post-hoc sensitivity analyses that are reported in the supplementary materials (Table S6, S7). In brief, model coefficients were largely robust to sensitivity analyses assuming two scenarios of data being missing: Either due to higher depression scores or due to higher psychological quality of life scores in the non-completer fraction (missing not at random). Follow-up data were not imputed due to a high rate of missingness. Independent sample *t*-tests and chi-square tests were used to test for group differences in demographics and pre-intervention scores between completers and non-completers.

For multiple imputations in the package *mice* (Version 3.14.0) [31], 508 variables were imputed in 25 data sets using predictive mean matching and a maximum of five iterations. As predictors for the imputation model we included WHOQOL-BREF and depression scores at baseline and 12 weeks, intervention group, and sociodemographic variables. A complete list of the included predictors are in the supplementary materials. Linear mixed effects models were fitted to each one of the 25 datasets using maximum likelihood estimation for model selection and restricted maximum likelihood for final parameter estimation. Model selection was accomplished by model comparisons based on the Akaike Information Criterion (AIC). Each WHOQOL-BREF domain served as the dependent variable in four separate models. Group, time, and the group * time interaction were entered as fixed effects. Random effects were calculated for each subject. Fixed and random effects coefficients of each data set were then pooled using Rubin’s rule [32]. Model coefficients are reported with their respective 95% CIs, *t*-values and *p*-values based on Satterwhite’s method for the degrees of freedom. Standardized effect sizes were calculated by dividing the model-based between-group differences by the square root of the residual variance and the random effects variance combined [33]. Effect sizes of 0.2 are considered small, 0.5 and larger as moderate, and larger than 0.8 as large [34]. Models fitted to the ITT sample were re-analyzed with the completer sample. To analyze the 24-week follow-up data, paired *t*-tests were calculated to assess the change from baseline within subjects per group. Bonferroni corrections were applied. Results of a post-hoc power analysis are reported in the supplementary materials.

In exploratory analyses of the ITT and completer sample, we added baseline depression severity, gender, age, relationship status, children, and employment status to the models. We further analyzed how change in depression severity (difference between BDI-II score at 12 weeks and baseline) related to quality of life ratings at 12 weeks in the completer sample. Additionally, we compared WHOQOL-BREF scores at 12 weeks between remitted and non-remitted participants (BDI-II score ≦ 10).

## Results

### Baseline data and attrition

817 prospective participants expressed interest in the study, of which 322 withdrew before the inclusion interview. Of the remaining 495 interviewees, 94 did not meet inclusion criteria, while 401 participants were included. At 24 weeks, 159 (39%) of the participants had completed the WHOQOL-BREF (65 (43%) in the guided intervention, 62 (41%) in the unguided intervention and 29 (29%) in the control condition. The attrition rate at 12 weeks differed significantly between intervention and control groups (χ^2^(2) = 36.3, *p* < 0.001). Baseline characteristics of the sample are reported in Table 1. Twenty-one percent of participants reported being in psychotherapeutic treatment at baseline with no significant differences between groups (χ^2^(2) = 1.5, *p* = 0.47) compared to 9.7% being in treatment at 12 weeks; 23 (5.7%) participants reported being in treatment both at baseline and at 12 weeks. In the intervention groups, 4% (guided) and 3.3% (unguided) of the participants started psychotherapy during the trial as opposed to 24% in the control condition. Table S1 shows the comparisons of baseline data between completers and non-completers. Baseline quality of life scores were significantly lower in the physical health and psychological health domains for completers compared to non-completers (both *p* < 0.05, Table S1).

**Table 1:** Sociodemographic and use of treatment data of the study sample

Characteristic	Guided intervention, *n* = 151	Unguided intervention, *n* = 150	Control group, *n* = 100	Total sample, *N* = 401
**Gender,** ***n*** **(%)**				
Female	126 (83.4)	126 (84.0)	81 (81.0)	333 (83.0)
Male	25 (16.6)	24 (16.0)	19 (19.0)	68 (17.0)
Age, *M* (*SD*)	38.1 (10.7)	36.6 (10.8)	36.2 (11.9)	37.1 (11.0)
**Relationship status,** ***n*** **(%)**				
Married/ partnered	54 (35.8)	33 (22.0)	52 (52.0)	139 (34.7)
No partner (divorced, separated, widowed)	19 (12.6)	8 (5.3)	19 (19.0)	46 (11.5)
Single	68 (45.0)	75 (50.0)	26 (26.0)	169 (42.1)
Not reported	10 (6.6)	34 (22.7)	3 (3.0)	47 (11.7)
**Children,** ***n*** **(%)**				
Yes	31 (20.5)	33 (22.0)	11 (11.0)	75 (18.7)
No	89 (58.9)	99 (66.0)	37 (37.0)	225 (56.1)
Not reported	31 (20.5)	18 (12.0)	52 (52.0)	101 (25.2)
**Professional training,** ***n*** **(%)**				
Still in training	11 (7.3)	6 (4.0)	16 (16)	33 (8.2)
Apprenticeship	28 (18.5)	19 (12.7)	25 (25)	72 (18.0)
Advanced vocational training	17 (11.3)	15 (10.0)	9 (9)	41 (10.2)
College or post-graduate degree	39 (25.8)	45 (30.0)	30 (30)	114 (28.4)
No training	15 (9.9)	18 (12.0)	8 (8)	41 (10.2)
Other	1 (0.7)	0 (0.0)	8 (8)	9 (2.2)
Not reported	40 (26.5)	47 (31.3)	4 (4)	91 (22.7)
**Employment,** ***n*** **(%)**				
Yes	85 (56)	90 (60)	59 (59)	234 (58)
In school/training	12 (8)	6 (4)	25 (25)	43 (11)
No/other	7 (4.6)	3 (2)	14 (14	67 (21)
Not reported	47 (31)	51 (34)	2 (2)	100 (25)
**Current psychotherapy at 12 weeks,** ***n*** **(%)**				
Yes	10 (6.6)	12 (8.0)	17 (17)	39 (9.7)
No	120 (79.5)	104 (69.3)	38 (38)	262 (65.3)
Not reported	21 (13.9)	34 (22.7)	45 (45)	100 (24.9)
**Start of psychotherapy during intervention**, ***n*** **(%)**				
Yes	6 (4.0)	5 (3.3)	14 (14)	25 (6.2)
No	124 (82.1)	111 (74.0)	41 (41)	276 (68.8)
Not reported	21 (13.9)	34 (22.7)	45 (45)	100 (24.9)
**Antidepressants at baseline,** ***n*** **(%)**				
Yes	45 (29.8)	25 (16.7)	25 (25)	95 (23.7)
No	106 (70.2)	125 (83.3)	75 (75)	306 (76.3)
Not reported	0 (0.0)	0 (0.0)	0 (0)	0 (0)
**Antidepressants at 12 weeks,** ***n*** **(%)**				
Yes	26 (17.2)	40 (26.7)	14 (14)	80 (20)
No	125 (82.8)	110 (73.3)	86 (86)	321 (80)
Not reported	0 (0.0)	0 (0.0)	0 (0)	0 (0)
**BDI at baseline,** ***M*** **(*SD*)**	30.1 (9.2)	30.5 (8.5)	30.9 (10.7)	30.5 (9.4)
**HRSD at baseline, *M*** **(*SD*)**	23.2 (6.3)	23.2 (6.8)	22.6 (6.8)	23.1 (6.6)
**Current major depressive episode,** ***n*** **(%)**				
Yes	143 (95)	132 (88)	78 (78)	353 (88)
No	8 (5)	18 (12)	22 (22)	48 (12)
**Lifetime major depressive episode**, ***n*** **(%)**				
Yes	94 (62)	103 (69)	63 (63)	260 (65)
No	57 (38)	47 (31)	37 (37)	141 (35)

*HRSD* Hamilton Rating Scale for Depression, *BDI* Beck Depression Inventory

### Results at the end of the intervention at 12 weeks

**Fig. 1:**
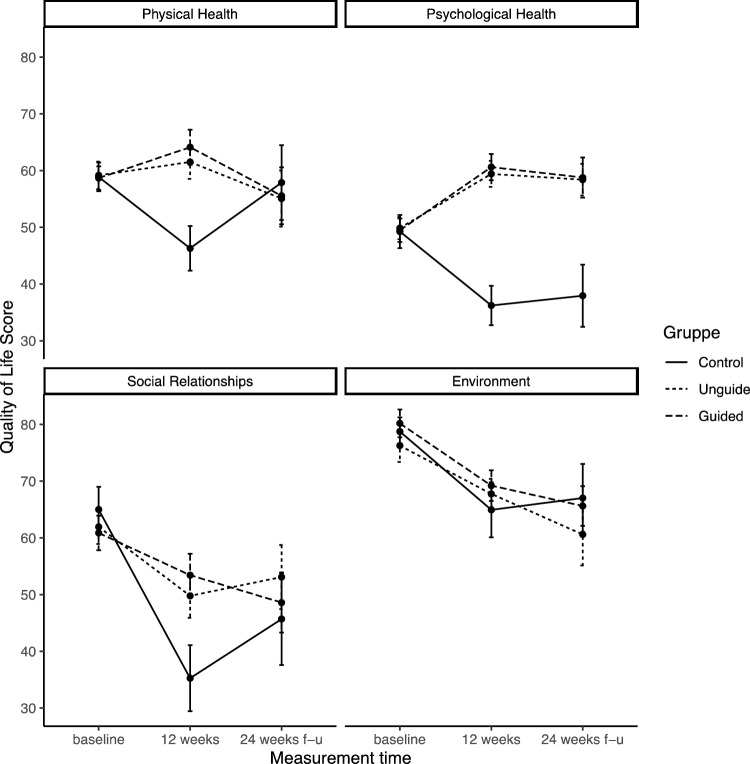
Means and 95% confidence intervals (error bars) of the completer sample for four WHOQOL-BREF domains across three measurement time points

Mean scores for all domains of the completer sample are shown in Fig. 1, and data for each subject are shown in Figure S1. Results of the linear mixed models of the ITT analysis including all 401 participants are shown in Table 2, and estimated marginal means based on the models are shown in Figure S2. There were significant group * time interactions at 12 weeks in three out of four quality of life domains: Physical health, psychological health, and social relationships. Receiving either the guided or the unguided intervention was associated with significantly higher quality of life scores in these domains compared to the control group with b*—estimates between 12.01 (*p* < 0.001) and 17.44 (*p* < 0.001) (Table 2). This translates into moderate to large effect sizes between 0.54 for the unguided intervention vs. controls on the social relationships scale and 1.30 for the guided intervention vs. control condition on the psychological health scale. However, the overall directions of change differed between domains: On the physical and psychological health scales, quality of life significantly improved for the guided and unguided intervention groups at 12 weeks, while for the control group it declined. On the scales social relationships and environment, quality of life declined for all groups over the course of the intervention. In the social relationships domain, it declined significantly more in the control group than in the intervention groups. In the environment domain, scores decreased equally for all groups between baseline and 12 weeks (b* = − 16.64, *p* < 0.001, effect size = − 0.81, 95% CI [− 0.99, − 0.62]) (Figs. 1 and S2, Table 2). In all models, random effects showed little to no influence on the fixed effects coefficients: Variance partition coefficients showed that between 0.0% and 3.8% of the total variance were attributable to differences between subjects. However, results from the models including random slopes are reported since they showed the best fit according to AICs. There were no significant differences between the guided and the unguided intervention in any domain.

**Table 2:** Results of the linear mixed model of the ITT sample for the four WHOQOL-BREF domains

Term	b*	*SE*	*t*	*p*	95% CI	Effect size [95% CI]
**Physical health**						
Intercept	58.89	1.50	39.20	< 0.001	[55.95, 61.84]	
Group: unguided	0.24	1.94	0.12	0.902	[− 3.57, 4.05]	
Guided	− 0.21	1.94	− 0.11	0.913	[− 4.01, 3.58]	
Time: 12 weeks	− 11.61	2.98	− 3.89	< 0.001	[− 17.46, − 5.76]	
Unguided × 12 weeks	12.01	3.49	3.44	0.001	[5.17, 18.85]	0.82 [0.42, 1.21]
Guided × 12 weeks	15.77	3.41	4.63	< 0.001	[9.09, 22.45]	1.04 [0.65, 1.42]
AIC 6624 VPC 3.2%						
**Psychological Health**						
Intercept	49.25	1.36	36.33	< 0.001	[46.59, 51.91]	
Group: unguided	0.61	1.75	0.35	0.728	[− 2.82, 4.05]	
Guided	0.23	1.75	0.13	0.897	[− 3.20, 3.65]	
Time: 12 weeks	− 7.15	2.54	− 2.81	0.006	[− 12.14, − 2.17]	
Unguided × 12 weeks	14.76	2.97	4.97	< 0.001	[8.93, 20.58]	1.13 [0.78, 1.49]
Guided × 12 weeks	17.44	2.94	5.93	< 0.001	[11.68, 23.20]	1.30 [0.95, 1.65]
AIC 6441						
VPC 0.0%						
**Social Relationships**						
Intercept	65.00	2.02	32.19	< 0.001	[61.04, 68.96]	
Group: unguided	− 3.03	2.61	− 1.16	0.246	[− 8.15, 2.08]	
Guided	− 4.13	2.60	− 1.59	0.113	[− 9.23, 0.97]	
Time: 12 weeks	− 28.23	3.74	− 7.54	< 0.001	[− 35.57, − 20.89]	
Unguided × 12 weeks	13.98	4.36	3.21	0.002	[5.44, 22.52]	0.54 [0.20, 0.9]
Guided × 12 weeks	19.63	4.47	4.39	< 0.001	[10.87, 28.40]	0.77 [0.41, 1.13]
AIC 7072 VPC 0.01%						
**Environment**						
Intercept	78.75	1.57	50.19	< 0.001	[75.67, 81.83]	
Group: unguided	− 2.28	2.03	− 1.12	0.261	[− 6.26, 1.69]	
Guided	1.44	2.02	0.71	0.475	[− 2.52, 5.41]	
Time: 12 weeks	− 16.64	3.19	− 5.21	< 0.001	[− 22.91, − 10.38]	
Unguided × 12 weeks	5.86	3.55	1.65	0.101	[− 1.10, 12.82]	0.19 [− 0.14, 0.59]
Guided × 12 weeks	4.12	3.68	1.12	0.266	[− 3.10, 11.33]	0.29 [− 0.03, 0.74]
AIC 6690 VPC 0.0%						

*AIC* Akaike Information Criterion, *CI* confidence interval, *IQR* Inter-quartile range, *Median VPC* Median variance portion coefficient

Results from the analyses of the completer sample revealed comparable results; however, effect sizes for the interaction terms (group * time) were trendwise larger (Table S2).

In exploratory analyses of the ITT data, we added baseline depression severity, gender, age, relationship status, children, and employment status to the models. Model estimates did not change after controlling for these factors. None of the factors showed any significant influence on WHOQOL ratings (Table S8).

Regarding the association of depression change with quality of life outcomes, we found a significant association between the change in depression severity and the change in quality of life scores for the physical (b* = 0.29, *p* < 0.001) and psychological health (b* = 0.34, *p* < 0.001) as well as the social relationships domain (b* = 0.39, *p* < 0.001). Patients who were remitted by 12 weeks (BDI-II scores ≦10) had significantly higher quality of life scores in the psychological health domain (*t*(350) = 2.3, *p* = 0.02) but not in the other domains.

Comparison of usage data between the guided and the unguided intervention group revealed that both completed a median amount of 10 modules (IQR_guided_ 9–11; IQR_unguided_ 8–11). In the guided intervention group, 86% of the participants completed more than six modules, in the unguided intervention group it was a fraction of 83%. Groups did not differ in the median amount of messages sent (both *Median* = 4, IQR_guide*d*_ 1–10; IQR_unguide*d*_ 0–13, *T* = 9852, *p* = 0.98) but in the amount of words written per message (*Median *_*guided*_ = 732 (IQR: 386–2149.5), *Median*_*unguided*_ = 1484 (IQR: 538–5829); *T* = 7528, *p* = 0.001) The guided intervention group made a mean number of 8 (*SD* = 3.6) calls during the trial with an average duration of 22 min (*SD* = 6.5).

### Results at 24-week follow-up after the intervention

Comparing the quality of life scores at baseline to scores at follow-up within groups, there were significant increases in psychological health scores for participants in both the guided and the unguided intervention (guided: *t*(64) =  − 4.1, adjusted *p* < 0.001; unguided: *t(*60) =  − 4.5, adjusted *p* < 0.001) (Table 3). Participants in the control group showed a significant decrease on the psychological health scale between baseline and follow-up (*t*(28) = 2.84, adjusted *p* < 0.05). The positive effect on physical health seen at 12 weeks was lost at 24-week follow-up. For the domains social relationships and environment, scores significantly decreased for almost all groups (Table 3).

**Table 3:** Results of paired *t*-tests for each group between baseline and 24-week follow-up for each WHOQOL-BREF domain in the completer sample

Group	Baseline		24 weeks		*t*	d*f*	*p* adjusted	Cohen’s *d*
	*M*	*SD*	*M*	*SD*				
**Physical Health**								
Guided	58.7	12.9	55.6	20.2	1.37	64	1	− 0.17
Unguided	59.2	15.1	55.1	19.5	0.58	60	1	− 0.07
Control	58.9	12.8	57.9	17.4	0.17	28	0.53	− 0.03
**Psychological Health**								
Guided	49.5	12.9	58.8	14.3	− 4.10	64	0.03	0.61
Unguided	49.9	12.2	58.4	11.0	− 4.49	60	< 0.001	0.61
Control	49.3	14.8	37.9	14.4	2.84	28	< 0.001	− 0.53
**Social Relationships**								
Guided	60.9	18.9	48.6	21.4	3.19	64	0.003	− 0.26
Unguided	61.9	18.8	53.1	22.2	2.38	60	0.06	− 0.43
Control	65	20.1	45.7	21.3	3.73	28	0.007	− 0.97
**Environment**								
Guided	80.2	15.3	65.6	14.1	6.29	64	< 0.001	− 0.46
Unguided	76.3	18.0	60.6	21.4	4.72	59	< 0.001	− 0.29
Control	78.8	12.6	67.0	15.8	4.42	28	< 0.001	− 0.76

## Discussion

In this article we report the results of a randomized controlled trial on the efficacy of a therapist-guided and an unguided version of the IBI *Selfapy* in improving the perceived quality of life in participants with depressive disorders. This is the analysis of a secondary outcome, the primary being depression severity. We found significant improvement of the quality of life in the domains physical health and psychological health for both the unguided and the guided intervention group compared to the control group over the course of the intervention (12 weeks). The benefits in the psychological health domain were maintained over another 12 weeks of follow-up post-intervention but not in the other domains. For the domains social relationships and environment, we observed a decline in reported quality of life over the course of the trial in all groups, while in the former the decline was more pronounced for the control group. The diverging pattern between domains could partly be explained by effects of the COVID-19 pandemic as we will discuss below. Given that *Selfapy* is conceptualized as a self-help program for patients with depression and has shown to improve depressive symptoms on several measures [24], it is plausible that this reduction of depression severity mainly entails improvement in the health-related quality of life domains but does not affect the social and environment domain. This association partly corresponds to meta-analytical data by Kolovos et al. (2016) who found that low depression severity is associated with high mental health-related quality of life but not physical or global measures of quality of life. This is also reflected in the association of change in depression severity and quality of life judgement at the end of the intervention in our exploratory analyses. Here, we showed that a decrease in depression severity is associated with an increase in quality of life mainly in the health domains and social relationships. The sustained positive effect on psychological health implies that the use of the IBI might not only reduce depression severity but could also ameliorate the subjective psychological quality of life beyond its use. However, psychological quality of life and depression severity are likely to be confounded in their measurement given that the WHOQOL-BREF items for this domain, such as “How well are you able to concentrate?” or “ How often do you have negative emotions, such as blue mood, despair, anxiety or depression?” directly capture aspects of depressive symptomatology.

Despite *Selfapy* addressing social relationships and communication, the social quality of life did not improve after baseline in any of the groups. This pattern of effects might be accounted for by the timing of data collection (between May 2019 and January 2021) that partly coincided with the rise of the COVID-19 pandemic and consecutive public health measures, such as lockdowns and restrictions on the number of social contacts. The first COVID-19 cases were registered in Germany by January 2020 and restrictions on public and private gatherings were in place from March to May 2020 and again from November 2021 until February 2022 [35]. It is conceivable that this affected at least partly how participants in our sample perceived their quality of life in the social and environmental domain.

Arguably, the treatment modality of telephone calls as opposed to video conferencing might have affected outcomes. To our knowledge, there is no systematic comparison of phone vs. video conferencing as a modality of treatment delivery. However, meta-analyses showing that phone-assisted vs. face-to-face delivery of psychotherapy are comparably effective suggest that the efficacy of the treatment is not dependent on seeing the therapist [36, 37].

Concurrent use of psychotherapy or antidepressants did not differ between groups at baseline and affected only a minority of the sample. We therefore argue that simultaneous use of in person psychotherapy did not have a major influence on our results. We found that participants in the control group were more likely to start psychotherapy during the trial. This suggests a higher demand for treatment in the waitlist control condition while this demand was less present in the intervention groups.

Contrary to previous meta-analyses [17], we did not find differences in efficacy between the guided and the unguided intervention. This could be due to the fact that the unguided group was given the chance to contact study personnel too which might have leveled effect sizes. Usage data showed that the unguided group sent longer messages in the chat function than the guided group which might have compensated for the call function unavailable to them. Another explanation might be a failure to establish rapport between the therapists and participants which would diminish efficacy of the guided intervention. However, the analysis of the therapeutic relationship was beyond the scope of this article.

Compared to population norms of the WHOQOL-BREF from an Australian sample published in 2006, mean quality of life scores at baseline were more than one standard deviation below the “norm” in the physical (norm: 73.5; *SD* = 18.1) and psychological (norm: 70.6, *SD* = 14.0) domain but within one standard deviation of the norm for social relationships (norm: 71.5; *SD* = 18.2) and for the environment (norm: 75.1; *SD* = 13.0) domain [38].

The majority of other randomized controlled trials in participants with diagnoses of MDD that offered guided IBIs with CBT elements did not report effects on quality of life. In a Finnish sample of young female university students (*N* = 124), a smartphone intervention adjunct to antidepressants neither improved depressive symptoms nor quality of life as measured by the EUROHIS-Qol, an adaptation of the WHOQOL-BREF [39]. Given that this sample—contrary to ours—was homogenous in age, gender, and education, these sociodemographic factors might have restricted the variance in quality of life judgements as they have been shown to influence the perception of quality of life independently from depression severity [4]. Furthermore, the instrument EUROHIS-Qol is a short form of the WHOQOL-BREF and therefore might be less sensitive to differential changes in the separate domains as it averages across them [40]. Furthermore, the Finnish intervention differed in several aspects. Its duration was 30% shorter than *Selfapy* and it offered only limited possibilities to contact a therapist. The chat function was described as “asynchronous” and phone calls were made only in exceptional circumstances. However, given that the unguided and guided intervention in our trial hardly differed in their outcome, the availability of therapist support via phone might not have been crucial for the differences. Another trial including participants with MDD showed comparable quality of life increases (*d* = 0.8) for both a blended treatment approach (behavioral activation face to face plus IBI) and full face-to-face behavioral activation in pre- to post-comparisons [41]. The authors emphasize that the blended treatment and the face-to-face treatment show comparable efficacy, while at the same time, the blended treatment has a much higher scalability. Other trials testing comparable IBIs for depressive symptoms that did not require diagnoses of MDD as an inclusion criterion or excluded MDD diagnoses altogether, did not show any effects on quality of life measures [42, 43]. So far, published studies on IBI interventions are too heterogeneous in study design, intervention, sample, and outcome to draw conclusions on the effects on quality of life. Ultimately, the heterogeneous definitions of quality of life and the use of numerous instruments measuring complementary facets of the construct limit comparability [5]. However, since IBIs are a widely usable and cost-efficient tool to help patients in need it is worthwhile studying and dismantling their effects. To unlock their full potential, large studies are required in order to reveal what makes them effective and how they could be of optimal use.

### Limitations

Despite its strengths, several limitations should be addressed. Firstly, we assessed quality of life only with one measure. Secondly, the attrition rate for the 24-week follow-up period was high (WHOQOL-BREF data was missing from 61% as opposed to 25.9% at 12 weeks). Arguably, the emails received by participants in the waitlist condition containing instructions for non-specific exercises might have had an effect on the control groups’ outcome. However, we cannot quantify this within our study design. In case the instructions had a positive effect, the effect sizes of our outcomes might be underestimated. Lastly, our study compared against a waitlist control, which is known to yield rather large effect sizes. However, this also reflects the reality of care since most patients wait for longer periods until they commence face-to-face psychotherapy, while IBIs are readily available.

### Supplementary Information

Below is the link to the electronic supplementary material.

Supplementary file1 (DOCX 202 KB)
