# Supplementary material for: Evaluation of the internet-based intervention “Selfapy” in participants with unipolar depression and the impact on quality of life: a randomized, parallel group study
Source: Qual Life Res. 2024 Feb 25;33(5):1275–86. doi: 10.1007/s11136-024-03606-2 (PMC11045620; doi:10.1007/s11136-024-03606-2)
Supplement: Supplementary file 1 — Supplementary file1 (DOCX 202 KB) [file 11136_2024_3606_MOESM1_ESM.docx]

**Supplementary Information**

**Evaluation of the internet-based intervention *“Selfapy”* in participants with unipolar depression and the impact on quality of life: a randomized, parallel group study**

Schefft, Cora^1^, Krämer, Rico ^1^, Haaf, Raoul^1^, Jedeck, David ^2^, Schumacher, Anna ^3^, Köhler, Stephan ^1^

^1^ Department of Psychiatry and Neurosciences, Charité – Universitätsmedizin Berlin,

Campus Mitte, Berlin, Germany

^2^ Department of Psychology, Humboldt University of Berlin, Germany

^3^ Department of Psychology, Sigmund-Freud Privat Universität, Berlin, Germany

Corresponding author:

Cora Schefft

Email: cora.schefft@charite.de

*Table S1 Comparison of baseline data between completers and non-completers. Categories marital status and employment were dichotomized.*

|  | **Completer (number or *mean. SD*)** | | **non-completer**  **(number or *mean. SD*)** | **degrees of freedom** | **χ^2^/*t*-value** | ***p*-value** |
| --- | --- | --- | --- | --- | --- | --- |
| Gender | 240 female. 57 male | | 93 female. 11 male | 1 | 347 | ns |
| Age | 37.22 (10.78) | | 36.63 (11.74) | 168 | -044 | ns |
| marital status | 156 single. 102 partnered | | 59 single. 37 partnered | 1 | 0002 | ns |
| Children | 168 no. 55 yes | | 57 no. 20 yes | 1 | 0.01 | ns |
| Number of children | 0.5 (0.88) | | 0.52 (0.9) | 129 | 0.16 | ns |
| household | 1.4 (1) | | 1.3 (0.9) | 208 | -0.97 | ns |
| Number of children in household | 0.27 (0.72) | | 0.23 (0.5) | 247 | -0.62 | ns |
| employment | 19 employed. 41 unemployed | | 11 employed. 13 unemployed | 1 | 0.95 | ns |
| current psychotherapy at baseline | 69 yes. 228 no | | 19 yes. 85 no | 1 | 0.84 | ns |
| antidepressants at baseline | 68 yes. 228 no | | 27 yes. 77 no | 1 | 0.25 | ns |
| BDI | 30.20 (9) | | 31.16 (10.21) | 163 | 0.848 | ns |
| HRSD | 23.51 (6.55) | | 21.84 (6.47) | 182 | -2.263 | < .05 |
| WHOQOL physical health | 57.98 (13.63) | | 61.57 (13.53) | 181 | 2.323 | < .05 |
| WHOQOL psychological health | 48.71 (12.62) | | 52 (14.1) | 164 | 2.105 | < .05 |
| WHOQOL social relationships | 61.64 (19) | | 64.18 (19.73) | 174 | 1.140 | ns |
| WHOQOL environment | 77.84 (16.32) | | 79.87 (14.15) | 206 | 1.206 | ns |
| current major depressive episode | 263 yes. 34 no | 90 yes. 14 no | | 1 | 0.14 | ns |
| lifetime major depressive episode | 187 yes. 110 no | 75 yes. 31 no | | 1 | 1 | ns |

**Model choices for the imputation model**

Description of the predictor matrix:

- 129 predictors included in all prediction models (“Inlist”).
  - “Inlist” with predictor description and count: Group (total: 1), WHOQOL items 1-26 measured at baseline and 12 weeks (total: 52), BDI items 1-21 measured at baseline, 6 weeks and 12 weeks (total: 63). Baseline sociodemographic variables (total: 13): Gender, age, nationality, marital status, children, number of children, size of household , number of children, number of children in household, school and higher education, occupation.
- Beyond the (forced) predictors specified in “inlist”, predictors were included based on the selection algorithm of the quickpred() function implemented in *mice*. We specified a predicting variable to have a minimal proportion of 30% usable cases and the correlation between two variables or their pattern of missingness to be at least *r* = 0.2. The resulting distribution of predictors for WHOQOL data (items 1-26) at 12 weeks (“WHO_3_x”) was:

| WHOQOL ITEM | NUMBER OF PREDICTORS |
| --- | --- |
| WHO_3_1 | 147 |
| WHO_3_2 | 144 |
| WHO_3_3 | 146 |
| WHO_3_4 | 133 |
| WHO_3_5 | 140 |
| WHO_3_6 | 139 |
| WHO_3_7 | 145 |
| WHO_3_8 | 133 |
| WHO_3_9 | 133 |
| WHO_3_10 | 144 |
| WHO_3_11 | 142 |
| WHO_3_12 | 133 |
| WHO_3_13 | 133 |
| WHO_3_14 | 133 |
| WHO_3_15 | 133 |
| WHO_3_16 | 139 |
| WHO_3_17 | 140 |
| WHO_3_18 | 133 |
| WHO_3_19 | 148 |
| WHO_3_20 | 133 |
| WHO_3_21 | 137 |
| WHO_3_22 | 133 |
| WHO_3_23 | 133 |
| WHO_3_24 | 133 |
| WHO_3_25 | 133 |
| WHO_3_26 | 139 |

The additional predictors were drawn from the dataset according to the quickpred() algorithm and included items from the psychometric instruments displayed in the table below (originally published in (1))

Table S1 Assessment instruments of the trial

| **Assessment timeframe** | **Assessment instrument** |
| --- | --- |
| Pre (T1) | Self-assessment—questionnaires: BDI-II; QIDS-SR16; BAI; WHOQOL-BREF; SWOP-K9; WAI-SR; APOI; SASS.  Assessment by a therapist – Questionnaires:MINI; HRSD-24 |
| During (T2) | Self-assessment—questionnaires: BDI-II; QIDS-SR16; BAI; WAI-SR; APOI |
| Post (T3) | Self-assessment—questionnaires:BDI-II; QIDS-SR16; BAI; WHOQOL-BREF; SWOP-K9; WAI-SR; APOI; SASS.  Assessment by a therapist—questionnaire: HRSD-24 |

1. *Abbreviations*: *MINI*, Mini International Neuropsychiatric Interview; *BDI-II*, Beck Depression Inventory-II; *QIDS-SR16*, Quick Inventory of Depressive Symptomatology; *BAI*, Beck Anxiety Inventory; *WHOQOL-BREF*, WHO Quality of Life-BREF; *SWOP-K9*, Self-Efficacy, Optimism, and Pessimism Scale; *WAI-SR*, Working Alliance Inventory - Short Revised; *APOI*, Attitudes towards Psychological Online Interventions Questionnaire; *Bado*, basic documentation; *HRSD-24*, Hamilton Depression Scale; *SASS*, Social Activity Self-Assessment Scale

**Individual patient data of the completer sample**

Figure S1. Spaghetti plot of individual participant data on WHOQOL-BREF scales at baseline, 12 and 24 weeks.

**Estimated marginal means of the linear mixed effects models (ITT sample)**

Figure S2. Estimated marginal means and their 95% confidence intervals of the quality of life scores in the intention to treat sample for all four WHOQOL-BREF domains at baseline and at 12 weeks.

**Results of the completer sample at 12 weeks (numerical)**

Table S3. Results (numerical) of the completer sample at 12 weeks

| **Time** | **Domain** | **Group** | | | | | | | | |
| --- | --- | --- | --- | --- | --- | --- | --- | --- | --- | --- |
|  |  | **guided** | | | **unguided** | | | **control** | | |
|  |  | **n** | **mean** | **SD** | **n** | **mean** | **SD** | **n** | **mean** | **SD** |
| **12 weeks** |  | | | | | | | | | |
|  | **Physical Health** | 128 | 64.1 | 17.8 | 116 | 61.5 | 16.2 | 52 | 46.3 | 14.1 |
|  | **Psychological Health** | 128 | 60.6 | 13.2 | 116 | 59.4 | 12.5 | 52 | 36.2 | 12.5 |
|  | **Social Relationships** | 129 | 53.4 | 21.6 | 116 | 49.8 | 21.0 | 52 | 35.3 | 20.9 |
|  | **Environment** | 129 | 69.2 | 15.5 | 116 | 67.8 | 14.5 | 51 | 65.0 | 17.3 |

**Post-hoc power calculation**

We calculated repeated measures ANOVAs for each WHOQOL-BREF domain by pooling the results from 25 imputed data sets and obtained effect sizes (*eta*^2) for the group*time interactions we were interested in.

We then entered these *eta^2* effect sizes in a power calculation tool (Bulus, M. (2023). pwrss: Statistical Power and Sample Size Calculation Tools. R package version 0.3.1. <https://CRAN.R-project.org/package=pwrss>) specifying a Type I error rate of 0.05, a sample size of 401, the correlation between values at baseline and 12 weeks to be 0.5 and entered the obtained *eta^2* for the group*time interaction. Results are listed in the table below.

Table S4. Results of the effect size and post-hoc power and sample size calculation.

| Domain | **eta^2 interaction** | **Power of the interaction term** | **N to obtain 95% power in the interaction given the effect sizes** |
| --- | --- | --- | --- |
| Physical health | 0.034125 | 1 | 75 |
| Psychological health | 0.061151 | 1 | 42 |
| Social relationships | 0.034870 | 1 | 73 |
| Environment | 0.004892 | 0.87 | 526 |

**Linear mixed effects models of the completer sample**

Table S5. Results of the linear mixed effects models of the completer sample. Legend: AIC = Akaike Information Criterion. VPC= Variance Partition Coefficient (% variance explained by random effects)

| Model: Value ~ group * time + (0+time \| Subject) | Beta-estimate | t - value | p - value | 95% - CI lower bound | 95% - CI upper bound | effect size | 95% - CI lower bound | 95% - CI upper bound |
| --- | --- | --- | --- | --- | --- | --- | --- | --- |
| **Physical Health** | | | | | | | | |
| Intercept | 58.89 | 40.12 | 0.00 | 56.02 | 61.77 |  |  |  |
| Unguided | 0.27 | 0.14 | 0.89 | - 3.44 | 3.99 |  |  |  |
| Guided | - 0.21 | - 0.11 | 0.91 | - 3.92 | 3.50 |  |  |  |
| Time: 12 weeks | - 12.53 | - 4.95 | 0.00 | - 17.50 | - 7.57 |  |  |  |
| Unguided * 12 weeks | 14.97 | 4.81 | 0.00 | 8.87 | 21.08 | -1.04 | -1.39 | -0.69 |
| Guided * 12 weeks | 18.00 | 5.84 | 0.00 | 11.96 | 24.05 | -1.21 | -1.56 | -0.87 |
| AIC: 5744 | | | | | | | | |
| VPC: 3.27% | | | | | | | | |
| **Psychological Health** | | | | | | | | |
| Intercept | 49.25 | 37.96 | 0.00 | 46.71 | 51.79 |  |  |  |
| Unguided | 0.61 | 0.36 | 0.72 | - 2.67 | 3.89 |  |  |  |
| Guided | 0.23 | 0.13 | 0.89 | - 3.05 | 3.50 |  |  |  |
| Time: 12 weeks | - 13.03 | - 5.87 | 0.00 | - 17.38 | - 8.68 |  |  |  |
| Unguided * 12 weeks | 22.58 | 8.25 | 0.00 | 17.22 | 27.95 | -1.79 | -2.13 | -1.45 |
| Guided * 12 weeks | 24.17 | 8.91 | 0.00 | 18.85 | 29.48 | -1.88 | -2.22 | -1.54 |
| AIC: 5547 | | | | | | | | |
| VPC: 0.0% | | | | | | | | |
| **Social Relationships** | | | | | | | | |
| Intercept | 65.00 | 32.51 | 0.00 | 61.08 | 68.92 |  |  |  |
| Unguided | - 3.06 | - 1.18 | 0.24 | - 8.11 | 2.00 |  |  |  |
| Guided | - 4.13 | - 1.60 | 0.11 | - 9.18 | 0.92 |  |  |  |
| Time: 12 weeks | - 29.74 | - 8.68 | 0.00 | - 36.45 | - 23.02 |  |  |  |
| Unguided * 12 weeks | 17.59 | 4.16 | 0.00 | 9.31 | 25.87 | -0.73 | -1.06 | -0.39 |
| Guided * 12 weeks | 22.29 | 5.33 | 0.00 | 14.10 | 30.48 | -0.91 | -1.24 | -0.58 |
| AIC: 6160 | | | | | | | | |
| VPC: 0.75% | | | | | | | | |
| **Environment** | | | | | | | | |
| Intercept | 78.75 | 50.46 | 0.00 | 75.69 | 81.81 |  |  |  |
| Unguided | - 2.48 | - 1.23 | 0.22 | - 6.43 | 1.47 |  |  |  |
| Guided | 1.44 | 0.72 | 0.47 | - 2.50 | 5.39 |  |  |  |
| Time: 12 weeks | - 13.80 | - 5.14 | 0.00 | - 19.06 | - 8.54 |  |  |  |
| Unguided * 12 weeks | 5.28 | 1.60 | 0.11 | - 1.20 | 11.76 | -0.18 | -0.51 | 0.15 |
| Guided * 12 weeks | 2.81 | 0.86 | 0.39 | - 3.60 | 9.23 | -0.27 | -0.60 | 0.05 |
| AIC: 5802 | | | | | | | | |
| VPC: 0.0% | | | | | | | | |

**Sensitivity analyses**

Multiple imputations assume that the data are missing at random (MAR) or missing completely at random (MCAR). MAR means that missingness depends only on observed data. In our case, we assumed the data to be MAR since MCAR is *a priori* unlikely and inspection of our data showed that completers and non-completers differed on some measures at baseline, rendering MCAR implausible. However, it is feasible that parts of the underlying mechanism of missingness is MNAR (missing not at random), meaning that there is non-ignorable missing data. However, in our case, this can’t be statistically inferred from the data at hand.

We saw at baseline, that completers of the trial had significantly higher quality of life scores on the psychological and physical health scales. In case the mechanism of missingness is related to non-completers having a higher quality of life, the missingness of the data would be MNAR.

We therefore performed sensitivity analyses for the following scenarios that seem likely given the design and question of our study:

1. How would the model parameters change if the WHOQOL psychological health scores at 12 weeks were systematically higher for the non-completers compared to those of completers? 🡪 hypothetically reduced need for the intervention.
2. How would the model parameters change if the BDI-II scores at 12 weeks were systematically higher for the non-completers compared to those of completers? 🡪 High drop-out rate due to high depression severity.

We compared scenarios in which we shifted the imputed (missing) WHOQOL psychological health scores post imputation by delta = (0, +7, +14) points and BDI-II scores by delta = (0, +5, +10) and reran the analysis under these assumptions. Values were chosen since 14 and 10 are the respective standard deviations of the WHOQOL psychological health subscale and the BDI-II in the completer sample at 12 weeks, 7 and 5 are each half a standard deviation. The value of zero represents the data under the MCAR assumption. Delta is added to all imputed values, simulating that the missing values are systematically higher than under the MCAR assumption.

Table S6 shows the model data under the delta – shifted imputation for WHOQOL-BREF psychological health. Our conclusions do not hold if delta = +14, since the missing values in the control group are imputed to be much higher than under MCAR assumption and the control group was more affected by missing data. Therefore the effect is diminished.

Table S7 shows sensitivity analyses under the assumption of higher BDI-II scores in the non-completer sample with delta= (+5, +10). Adding delta did not lead to changes in the model parameters that were beyond the confidence intervals of our model under the MCAR assumption.

Table S6 Coefficients of the linear mixed models for the WHOQOL subscales for delta - adjustments 0, 7 or 14 to the WHOQOL psychological health scale at 12 weeks.. Values that alter the conclusion from the model used in the ITT analysis are printed in bold face.

|  | **Delta= 0** | | | **Delta = 7** | | | **Delta = 14** | | |
| --- | --- | --- | --- | --- | --- | --- | --- | --- | --- |
| Term | *b** | *p* | 95% CI | *b** | *p* | 95% CI | *b** | *p* | 95% CI |
| **Physical health** | | | | | | | | | |
| Intercept | 58.89 | < .001 | [55.94, 61.84] | 58.89 | < .001 | [55.97, 61.82] | 58.89 | < .001 | [56.00, 61.78] |
| Group Unguided | 0.27 | .888 | [-3.53, 4.08] | 0.27 | .887 | [-3.51, 4.05] | 0.27 | .886 | [-3.46, 4.01] |
| Group Guided | -0.21 | .913 | [-4.01, 3.59] | -0.21 | .912 | [-3.99, 3.56] | -0.21 | .911 | [-3.94, 3.52] |
| Time 12 weeks | -9.15 | .003 | [-14.58, -3.72] | -11.72 | .001 | [-17.27, -6.18] | -6.08 | .011 | [-10.64, -1.52] |
| Unguided × 12 weeks | 9.60 | .007 | [3.03, 16.16] | 12.07 | < .001 | [6.36, 17.78] | 8.65 | .002 | [3.27, 14.02] |
| Guided × 12 weeks | 13.01 | .001 | [6.29, 19.72] | 15.64 | < .001 | [8.80, 22.48] | 11.03 | < .001 | [5.28, 16.78] |
| **Psychological health** | | | | | | | | | |
| Intercept | 49.25 | < .001 | [46.56, 51.94] | 49.25 | < .001 | [46.60, 51.90] | 49.25 | < .001 | [46.14, 52.36] |
| Group Unguided | 0.61 | .730 | [-2.86, 4.08] | 0.61 | .727 | [-2.82, 4.04] | 0.61 | .766 | [-3.41, 4.63] |
| Group Guided | 0.23 | .898 | [-3.24, 3.69] | 0.23 | .897 | [-3.20, 3.65] | 0.23 | .912 | [-3.79, 4.24] |
| Time 12 weeks | -2.88 | .186 | [-7.12, 1.36] | -7.30 | .002 | [-11.72, -2.88] | 13.93 | < .001 | [9.64, 18.21] |
| Unguided × 12 weeks | 12.16 | < .001 | [6.91, 17.42] | 14.68 | < .001 | [9.41, 19.94] | **3.17** | **.259** | **[-2.33, 8.67]** |
| Guided × 12 weeks | 13.15 | < .001 | [7.85, 18.45] | 17.14 | < .001 | [12.02, 22.26] | **1.73** | **.538** | **[-3.77, 7.22]** |
| **Social Relationships** | | | | | | | | | |
| Intercept | 65.00 | < .001 | [61.02, 68.98] | 65.00 | < .001 | [61.06, 68.94] | 65.00 | < .001 | [61.03, 68.97] |
| Group Unguided | -3.06 | .245 | [-8.20, 2.09] | -3.06 | .239 | [-8.14, 2.03] | -3.06 | .243 | [-8.18, 2.07] |
| Group Guided | -4.13 | .116 | [-9.26, 1.01] | -4.13 | .112 | [-9.21, 0.95] | -4.13 | .114 | [-9.24, 0.99] |
| Time 12 weeks | -24.78 | < .001 | [-32.22, -17.35] | -27.38 | < .001 | [-33.88, -20.88] | -17.67 | .004 | [-26.79, -8.55] |
| Unguided × 12 weeks | 10.43 | .016* | [2.17, 18.68] | 13.27 | .001 | [5.45, 21.09] | **7.53** | **.115** | **[-1.54, 16.60]** |
| Guided × 12 weeks | 15.95 | .006 | [5.96, 25.94] | 18.69 | < .001 | [10.23, 27.15] | 10.86 | .041 | [1.13, 20.58] |
| **Environment** | | | | | | | | | |
| Intercept | 78.75 | < .001 | [75.67, 81.83] | 78.75 | < .001 | [75.69, 81.81] | 78.75 | < .001 | [75.75, 81.75] |
| Group Unguided | -2.48 | .222 | [-6.45, 1.49] | -2.48 | .219 | [-6.43, 1.47] | -2.48 | .210 | [-6.35, 1.40] |
| Group Guided | 1.44 | .476 | [-2.52, 5.41] | 1.44 | .473 | [-2.50, 5.39] | 1.44 | .465 | [-2.42, 5.31] |
| Time 12 weeks | -15.25 | < .001 | [-21.04, -9.46] | -15.41 | < .001 | [-21.17, -9.66] | -11.92 | < .001 | [-16.93, -6.92] |
| Unguided × 12 weeks | 4.85 | .116 | [-1.17, 10.86] | 5.17 | .098 | [-0.91, 11.25] | 2.94 | .356 | [-3.26, 9.14] |
| Guided × 12 weeks | 2.65 | .429 | [-3.87, 9.16] | 3.01 | .430 | [-4.32, 10.33] | -0.14 | .966 | [-6.55, 6.27] |

Table S7 Coefficients of the linear mixed models for the WHOQOL subscales for delta - adjustments 0, 5 or 10 to BDI-II scale at 12 weeks.

|  | **Delta= 0** | | | **Delta = 5** | | | **Delta = 10** | | |
| --- | --- | --- | --- | --- | --- | --- | --- | --- | --- |
| Term | *b** | *p* | 95% CI | *b** | *p* | 95% CI | *b** | *p* | 95% CI |
| **Physical health** | | | | | | | | | |
| Intercept | 58.89 | < .001 | [55.97, 61.81] | 58.89 | < .001 | [55.96, 61.82] | 58.89 | < .001 | [56.00, 61.79] |
| Group Unguided | 0.24 | .901 | [-3.54, 4.01] | 0.24 | .901 | [-3.55, 4.03] | 0.24 | .900 | [-3.50, 3.98] |
| Group Guided | -0.21 | .912 | [-3.98, 3.55] | -0.21 | .912 | [-3.99, 3.57] | -0.21 | .911 | [-3.94, 3.52] |
| Time 12 weeks | -10.63 | < .001 | [-15.67, -5.59] | -9.54 | .017 | [-16.15, -2.93] | -8.00 | .011 | [-13.51, -2.49] |
| Unguided × 12 weeks | 10.96 | .001 | [4.86, 17.07] | 10.17 | .004 | [3.73, 16.60] | 9.80 | .001 | [4.28, 15.32] |
| Guided × 12 weeks | 14.50 | < .001 | [8.80, 20.20] | 13.56 | < .001 | [6.85, 20.27] | 12.95 | < .001 | [7.45, 18.45] |
| **Psychological health** | | | | | | | | | |
| Intercept | 49.25 | < .001 | [46.59, 51.91] | 49.25 | < .001 | [46.62, 51.88] | 49.25 | < .001 | [46.56, 51.94] |
| Group Unguided | 0.61 | .728 | [-2.82, 4.05] | 0.61 | .725 | [-2.79, 4.01] | 0.61 | .731 | [-2.87, 4.09] |
| Group Guided | 0.23 | .897 | [-3.20, 3.65] | 0.23 | .896 | [-3.17, 3.62] | 0.23 | .899 | [-3.25, 3.70] |
| Time 12 weeks | -7.15 | .006 | [-12.14, -2.17] | -7.31 | .088 | [-14.56, -0.06] | -4.77 | .090 | [-9.97, 0.44] |
| Unguided × 12 weeks | 14.76 | < .001 | [8.93, 20.58] | 15.03 | < .001 | [8.42, 21.65] | 13.47 | < .001 | [8.16, 18.79] |
| Guided × 12 weeks | 17.44 | < .001 | [11.68, 23.20] | 17.28 | < .001 | [10.22, 24.33] | 15.83 | < .001 | [10.05, 21.60] |
| **Social Relationships** | | | | | | | | | |
| Intercept | 65.00 | < .001 | [61.03, 68.97] | 65.00 | < .001 | [61.09, 68.91] | 65.00 | < .001 | [61.01, 68.99] |
| Group Unguided | -3.03 | .247 | [-8.16, 2.09] | -3.03 | .240 | [-8.08, 2.02] | -3.03 | .249 | [-8.19, 2.12] |
| Group Guided | -4.13 | .114 | [-9.24, 0.98] | -4.13 | .109 | [-9.17, 0.91] | -4.13 | .116 | [-9.27, 1.01] |
| Time 12 weeks | -28.27 | < .001 | [-34.27, -22.26] | -29.25 | < .001 | [-37.62, -20.88] | -26.02 | < .001 | [-34.21, -17.82] |
| Unguided × 12 weeks | 13.12 | .001 | [5.46, 20.78] | 14.41 | .003 | [5.66, 23.16] | 11.92 | .005 | [3.80, 20.05] |
| Guided × 12 weeks | 19.20 | < .001 | [11.72, 26.69] | 20.28 | < .001 | [11.26, 29.29] | 17.48 | < .001 | [9.49, 25.48] |
| **Environment** | | | | | | | | | |
| Intercept | 78.75 | < .001 | [75.69, 81.81] | 78.75 | < .001 | [75.64, 81.86] | 78.75 | < .001 | [75.72, 81.78] |
| Group Unguided | -2.28 | .258 | [-6.23, 1.67] | -2.28 | .267 | [-6.31, 1.74] | -2.28 | .254 | [-6.20, 1.63] |
| Group Guided | 1.44 | .473 | [-2.50, 5.38] | 1.44 | .481 | [-2.57, 5.46] | 1.44 | .469 | [-2.46, 5.35] |
| Time 12 weeks | -15.74 | < .001 | [-21.45, -10.04] | -18.56 | < .001 | [-25.70, -11.41] | -15.25 | .001 | [-21.96, -8.54] |
| Unguided × 12 weeks | 5.24 | .092 | [-0.82, 11.31] | 6.81 | .076 | [-0.42, 14.04] | 4.63 | .232 | [-2.74, 12.01] |
| Guided × 12 weeks | 3.11 | .397 | [-3.97, 10.20] | 5.31 | .197 | [-2.45, 13.07] | 3.11 | .435 | [-4.50, 10.71] |

**Models with additional predictors**

*Table S8. ITT models including additional predictors and Beck depression inventory (BDI-II) change scores.*

| Term | *b** | *SE* | *t* | *df* | *p* | 95% CI |
| --- | --- | --- | --- | --- | --- | --- |
| **Physical health** |  |  |  |  |  |  |
| Intercept | 59.35 | 4.02 | 14.76 | 377.63 | < .001 | [51.47, 67.24] |
| Group Unguided | 0.18 | 2.00 | 0.09 | 769.10 | .929 | [-3.74, 4.09] |
| Group Guided | -0.21 | 1.97 | -0.11 | 772.67 | .914 | [-4.08, 3.66] |
| Time 12 weeks | -11.61 | 2.98 | -3.89 | 76.48 | < .001 | [-17.46, -5.76] |
| BDI-II baseline | -0.05 | 0.07 | -0.71 | 256.19 | .479 | [-0.18, 0.09] |
| Male gender | 1.64 | 1.56 | 1.05 | 600.62 | .295 | [-1.43, 4.70] |
| Age | -0.06 | 0.06 | -1.03 | 405.39 | .302 | [-0.17, 0.05] |
| Partner vs. no partner | 0.16 | 1.32 | 0.12 | 354.69 | .901 | [-2.43, 2.76] |
| Children yes vs. no | 1.82 | 1.57 | 1.16 | 219.44 | .247 | [-1.26, 4.91] |
| In training vs. unemployed | 2.92 | 3.15 | 0.93 | 149.92 | .355 | [-3.25, 9.09] |
| (Self-)/employed vs. unemployed | 2.57 | 2.72 | 0.95 | 162.07 | .346 | [-2.76, 7.90] |
| Other employment vs. unemployed | 0.39 | 5.37 | 0.07 | 175.70 | .942 | [-10.14, 10.92] |
| Unguided × 12 weeks | 12.01 | 3.49 | 3.44 | 118.30 | .001 | [5.18, 18.84] |
| Guided × 12 weeks | 15.77 | 3.41 | 4.63 | 132.46 | < .001 | [9.10, 22.45] |
| **Psychological health** |  |  |  |  |  |  |
| Intercept | 48.03 | 3.54 | 13.57 | 331.21 | < .001 | [41.09, 54.97] |
| Group Unguided | 0.71 | 1.81 | 0.39 | 761.66 | .696 | [-2.84, 4.25] |
| Group Guided | 0.29 | 1.79 | 0.16 | 767.35 | .870 | [-3.21, 3.79] |
| Time 12 weeks | -7.15 | 2.54 | -2.81 | 99.72 | .006 | [-12.14, -2.17] |
| BDI-II baseline | -0.01 | 0.06 | -0.23 | 272.13 | .818 | [-0.13, 0.10] |
| Male gender | 1.01 | 1.36 | 0.74 | 540.77 | .457 | [-1.65, 3.68] |
| Age | -0.01 | 0.05 | -0.12 | 338.17 | .903 | [-0.10, 0.09] |
| Partner vs. no partner | -0.05 | 1.12 | -0.04 | 431.86 | .965 | [-2.24, 2.14] |
| Children yes vs. no | 1.79 | 1.36 | 1.32 | 212.81 | .189 | [-0.87, 4.46] |
| In training vs. unemployed | 2.17 | 2.73 | 0.80 | 142.61 | .427 | [-3.18, 7.53] |
| (Self-)/employed vs. unemployed | 1.13 | 2.30 | 0.49 | 183.71 | .624 | [-3.37, 5.63] |
| Other employment vs. unemployed | 1.65 | 4.33 | 0.38 | 290.57 | .704 | [-6.85, 10.14] |
| Unguided × 12 weeks | 14.76 | 2.97 | 4.96 | 174.83 | < .001 | [8.93, 20.59] |
| Guided × 12 weeks | 17.44 | 2.94 | 5.93 | 185.77 | < .001 | [11.67, 23.20] |
| **Social relationships** |  |  |  |  |  |  |
| Intercept | 67.30 | 4.57 | 14.72 | 285.28 | < .001 | [58.33, 76.26] |
| Group Unguided | -3.50 | 2.77 | -1.26 | 747.45 | .208 | [-8.93, 1.94] |
| Group Guided | -4.54 | 2.76 | -1.65 | 749.67 | .100 | [-9.94, 0.87] |
| Time 12 weeks | -28.23 | 3.75 | -7.54 | 104.47 | < .001 | [-35.57, -20.89] |
| BDI-II baseline | 0.02 | 0.07 | 0.30 | 335.61 | .763 | [-0.11, 0.15] |
| Male gender | 0.88 | 2.04 | 0.43 | 584.36 | .667 | [-3.11, 4.87] |
| Age | -0.00 | 0.07 | -0.01 | 342.00 | .992 | [-0.15, 0.14] |
| Partner vs. no partner | -0.25 | 1.75 | -0.14 | 298.32 | .888 | [-3.68, 3.18] |
| Children yes vs. no | -0.72 | 2.12 | -0.34 | 167.36 | .736 | [-4.88, 3.44] |
| In training vs. unemployed | -3.75 | 4.16 | -0.90 | 133.96 | .369 | [-11.91, 4.41] |
| (Self-)/employed vs. unemployed | -1.69 | 3.71 | -0.46 | 121.74 | .649 | [-8.96, 5.58] |
| Other employment vs. unemployed | -7.39 | 6.88 | -1.07 | 195.34 | .284 | [-20.88, 6.10] |
| Unguided × 12 weeks | 13.98 | 4.36 | 3.21 | 192.74 | .002 | [5.44, 22.53] |
| Guided × 12 weeks | 19.63 | 4.48 | 4.39 | 158.20 | < .001 | [10.86, 28.41] |
| **Environment** |  |  |  |  |  |  |
| Intercept | 79.45 | 4.55 | 17.46 | 148.06 | < .001 | [70.53, 88.37] |
| Group Unguided | -2.79 | 2.11 | -1.32 | 736.85 | .187 | [-6.92, 1.35] |
| Group Guided | 1.01 | 2.08 | 0.48 | 745.99 | .629 | [-3.08, 5.09] |
| Time 12 weeks | -16.65 | 3.20 | -5.21 | 72.57 | < .001 | [-22.91, -10.38] |
| BDI-II baseline | -0.05 | 0.07 | -0.71 | 283.66 | .476 | [-0.18, 0.08] |
| Male gender | 0.18 | 1.57 | 0.11 | 549.87 | .910 | [-2.90, 3.26] |
| Age | -0.01 | 0.06 | -0.23 | 370.82 | .816 | [-0.12, 0.10] |
| Partner vs. no partner | -0.35 | 1.46 | -0.24 | 158.67 | .809 | [-3.21, 2.50] |
| Children yes vs. no | 0.33 | 1.67 | 0.19 | 141.11 | .846 | [-2.95, 3.61] |
| In training vs. unemployed | 0.53 | 3.74 | 0.14 | 68.28 | .887 | [-6.80, 7.87] |
| (Self-)/employed vs. unemployed | 2.23 | 3.46 | 0.65 | 58.03 | .521 | [-4.54, 9.01] |
| Other employment vs. unemployed | -4.54 | 6.03 | -0.75 | 93.12 | .453 | [-16.35, 7.27] |
| Unguided × 12 weeks | 5.86 | 3.55 | 1.65 | 143.59 | .101 | [-1.11, 12.83] |
| Guided × 12 weeks | 4.12 | 3.69 | 1.12 | 115.85 | .266 | [-3.11, 11.34] |

All hyperlinks were last accessed on the 25th of November 2023

**References**

1. Krämer R, Köhler S. Evaluation of the online-based self-help programme "Selfapy" in patients with unipolar depression: study protocol for a randomized, blinded parallel group dismantling study. Trials. 2021;22(1):264.
